# Serial Changes in Routine Inflammatory, Hematologic, and Renal Laboratory Markers in Adults Hospitalized with Clinically Recorded Sepsis: A Longitudinal Observational Cohort Study

**DOI:** 10.3390/jcm15218246

**Published:** 2026-10-23

**Authors:** Andra Rotea, Bogdan Rotea, Mădălina Suba, Ovidiu Roșca, Bogdan Cerbu, Ileana Enătescu, Mirabela Dima, Emil Radu Iacob, Adrian Gluhovschi, Daniela Iacob

**Affiliations:** 1 Doctoral School, Victor Babeș University of Medicine and Pharmacy, 300041 Timișoara, Romania; andra.alecu@umft.ro (A.R.); bogdan.cerbu@umft.ro (B.C.); 2 Center for Multidisciplinary Research and Management in Pediatric Surgical Conditions, Iosif Nemoianu Street No. 2, 300011 Timișoara, Romania; enatescu.ileana@umft.ro (I.E.); dima.mirabela@umft.ro (M.D.); radueiacob@umft.ro (E.R.I.); iacob.daniela@umft.ro (D.I.); 3 Methodological and Infectious Diseases Research Center, Department of Infectious Diseases, Victor Babeș University of Medicine and Pharmacy, Eftimie Murgu Square No. 2, 300041 Timișoara, Romania; madalina.suba@umft.ro (M.S.); ovidiu.rosca@umft.ro (O.R.); 4 Dr. Victor Babeș Clinical Hospital of Infectious Diseases and Pneumophthisiology, 300310 Timișoara, Romania; 5 Department XIII, Discipline of Infectious Diseases, Victor Babeș University of Medicine and Pharmacy, Eftimie Murgu Square No. 2, 300041 Timișoara, Romania; 6 Discipline of Neonatology, Victor Babeș University of Medicine and Pharmacy, 300041 Timișoara, Romania; 7 Department of Pediatric Surgery and Orthopedics, Victor Babeș University of Medicine and Pharmacy, Eftimie Murgu Square No. 2, 300041 Timișoara, Romania; 8 Department XII, Discipline of Obstetrics and Gynecology, Victor Babeș University of Medicine and Pharmacy, 300041 Timișoara, Romania; adigluhovschi@yahoo.com

**Keywords:** sepsis, procalcitonin, C-reactive protein, longitudinal analysis, biomarkers, mixed-effects models, creatinine, platelet count

## Abstract

**Background/Objectives**: Serial laboratory measurements may capture within-episode changes not represented by a single baseline value, while routine markers from different biological domains may not evolve concordantly. **Methods**: We retrospectively analyzed 100 hospital admission episodes in which the source infection field explicitly recorded sepsis. A complete source-record re-audit confirmed sepsis wording in all 100 episodes and identified no severe-infection-only subgroup. Because complete SOFA components were unavailable, Sepsis-3 criteria were not retrospectively adjudicated. Procalcitonin (PCT), C-reactive protein (CRP), white blood cell count (WBC), platelet count, and serum creatinine were evaluated across three ordered recorded assessment points using random-intercept linear mixed-effects models, with Holm correction across five primary global tests and generalized estimating equations as robustness analyses. **Results**: PCT geometric means decreased from 0.925 to 0.306 ng/mL (Holm-adjusted *p* = 0.000052), CRP from 104.2 to 36.0 mg/L (*p* < 0.001), and serum creatinine from 1.49 to 1.10 mg/dL (*p* = 0.000074). Platelet count increased on the additive scale (*p* = 0.000237) but not after logarithmic transformation, whereas the full-cohort primary WBC model was non-significant (*p* = 0.0743). **Conclusions**: Routine laboratory markers showed heterogeneous cohort-level longitudinal patterns, supporting marker-specific rather than uniform interpretation of serial measurements.

## 1. Introduction

Sepsis is defined as life-threatening organ dysfunction caused by a dysregulated host response to infection and is characterized by substantial biological and clinical heterogeneity [1]. Alterations in innate and adaptive immunity, inflammation, coagulation, endothelial function, metabolism, and organ-specific responses vary among patients and over time [2]. This heterogeneity provides a rationale for evaluating serial laboratory measurements rather than relying exclusively on isolated values.

The burden of sepsis is substantial but varies with population, case ascertainment, and health-system setting. The Global Burden of Disease 2017 analysis estimated 48.9 million incident sepsis cases and 11.0 million sepsis-related deaths worldwide [3]; an updated GBD analysis also documented considerable regional variation through 2021 [4]. Romanian epidemiological information remains dominated by institution-based studies, which should not be interpreted as population-level national incidence. At a major emergency hospital in Bucharest, Mușat et al. identified 12,089 patients with recorded sepsis among 913,405 admissions during 2007–2024 (1.32% of admissions at that hospital) [5]. In southwestern Romania, Grigorescu et al. characterized 184 patients with severe infections recruited under Sepsis-2-era definitions [6]. These cohorts reflect different populations and definitions, underscoring the value of carefully described Romanian hospital-based datasets and the need to avoid extrapolating single-center estimates to the whole country.

C-reactive protein (CRP) and procalcitonin (PCT) are among the most frequently used inflammatory biomarkers in severe infection and sepsis, but isolated concentrations have limited ability to represent the full host response or subsequent clinical course [7]. Serial studies have therefore examined their temporal behavior. Schupp et al. evaluated 349 patients with sepsis or septic shock and demonstrated substantial changes in CRP and PCT during the course of illness, while discrimination for 30-day mortality remained limited [8]. Orfanu et al. prospectively evaluated 77 septic patients and described distinct early kinetic behavior across several inflammatory biomarkers [9].

The prognostic implications of serial biomarker measurements have also been studied in Romanian cohorts. Varga et al. retrospectively evaluated 138 patients with severe infections using serial PCT, CRP, and lactate measurements and focused on their association with 30-day mortality [10]. More recent work has expanded toward multidimensional trajectory analysis. Xu et al. evaluated 17 nutritional, immune, and inflammatory biomarkers at five time points in 712 adults with burn sepsis and demonstrated substantial heterogeneity in longitudinal biomarker patterns [11].

Recent longitudinal studies have also used routine laboratory data to characterize temporal biological changes without necessarily constructing prognostic models. In a 2026 prospective cohort of 389 patients with sepsis, Turcato et al. used repeated routine laboratory measurements to characterize longitudinal changes in albumin homeostasis, illustrating the value of serial clinical laboratory data for describing evolving pathophysiology [12].

A substantial proportion of the existing literature nevertheless emphasizes mortality prediction, risk stratification, specialized biomarkers, or predefined biological phenotypes. In routine care, inflammatory, hematologic, and renal laboratory variables are interpreted simultaneously, although they reflect different biological domains and may not show concordant temporal behavior. Moreover, apparent longitudinal changes can depend on analytical scale, model specification, incomplete repeated measurements, and data-quality decisions. These issues are especially relevant in real-world observational datasets in which testing is determined by clinical care rather than by a fixed research sampling schedule.

The anatomical source of infection may contribute additional heterogeneity. Peters-Sengers et al. demonstrated source-specific differences across multiple host-response pathways in critically ill patients with sepsis [13]. Whether analogous differences are detectable using routinely collected laboratory markers in less extensively phenotyped cohorts remains uncertain.

The primary aim of this study was therefore to characterize within-episode longitudinal changes in routine inflammatory, hematologic, and renal laboratory markers across serial recorded assessments in adults hospitalized with clinically recorded sepsis. PCT, CRP, WBC, platelet count, and serum creatinine were prespecified as the five primary longitudinal laboratory variables. The central question was whether these routine markers showed concordant or heterogeneous cohort-level longitudinal behavior and whether the observed effects were robust to alternative statistical specifications.

## 2. Materials and Methods

### 2.1. Study Design and Reporting

This retrospective longitudinal observational cohort analysis included adult hospital admission episodes with clinically recorded sepsis between 9 December 2024 and 22 December 2025. The analysis used deidentified source data and the manuscript was prepared in accordance with the Strengthening the Reporting of Observational Studies in Epidemiology (STROBE) framework for cohort studies. Institutional authorization and ethics review are described in the Institutional Review Board Statement.

For the present analysis, all 100 hospital admission episodes contained in the primary three-assessment dataset were retained in the inferential cohort. Cohort construction was based on source-dataset membership and the availability of the internally coherent three-assessment data structure rather than on observed study outcomes.

### 2.2. Source Data and Construction of the Analytic Cohort

The available adult source data comprised 130 source rows from two independently maintained datasets. The primary three-assessment dataset contained 100 hospital admission episodes with measurements recorded at M0, M2, and M3 and formed the primary inferential cohort. A secondary dataset contained 30 source rows with M0 and M2 measurements but no M3 measurements. The two datasets were not pooled because cross-dataset harmonization of several laboratory variables could not be established with sufficient confidence from the available source documentation. In particular, marked scale differences were present for WBC and platelet count, and additional unit/type heterogeneity affected other variables. No assumed global conversion factors were applied.

The unit of analysis was the hospital admission episode. The primary dataset contained 100 distinct admission records. Definitive patient-level identifiers were unavailable in the deidentified analytic dataset. Three pairs of episodes shared the same combination of initials, age, and sex and could represent repeated admissions by the same individual, but identity could not be established; these episodes therefore remained separate in the primary analysis. This uncertainty is addressed as a limitation rather than treated as confirmed duplicate-person linkage.

A complete source-record re-audit showed that the infection-source field explicitly contained “Sepsis” in all 100 primary admission episodes; none was labeled as severe infection without sepsis. The treating department was an infectious-diseases clinical service staffed by specialist and consultant infectious-diseases physicians. According to clinical-site confirmation, sepsis was diagnosed clinically during routine care; the source research spreadsheet does not identify the individual diagnosing physician or retain a uniform, patient-level diagnostic algorithm. Accordingly, this study concerns clinically diagnosed, source-recorded sepsis rather than a retrospectively Sepsis-3-adjudicated cohort. Complete SOFA components were not available for verification of Sepsis-3 organ-dysfunction criteria. Because no severe-infection-only group was recorded in this dataset, a sepsis-versus-severe-infection comparison was not applicable.

Underlying diseases were re-audited from the source comorbidity field for all 100 admission episodes and are presented at admission-episode level in Supplementary Table S7. Immune status was not a prespecified eligibility criterion. Although the source extract includes HIV infection, hematologic malignancy or its history, and solid neoplasm/tumor mentions, it does not systematically capture active oncologic treatment, immunosuppressive medications, recent chemotherapy, transplant history, systemic corticosteroid exposure, or neutropenia. Therefore, no patient without an explicit immunocompromising condition was automatically designated immunocompetent, and the cohort was not dichotomized into definitively immunocompetent and immunocompromised groups. As a post hoc reviewer-requested sensitivity analysis, we excluded six episodes with explicitly recorded HIV infection (*n* = 3) or hematologic malignancy/history (*n* = 3); results are reported in Supplementary Table S8. Cohort selection and assessment-point definitions are summarized in Figure 1.

### 2.3. Serial Assessment Points

Laboratory measurements were recorded at three ordered assessment points designated M0, M2, and M3. The original source-data explanation explicitly defined admission measurements as the first available measurement (“prima ocazie—prima măsurare”); M0 therefore represents the first available admission measurement. Exact analyte-specific specimen timestamps and exact elapsed times for M2 and M3 were not retained in the research spreadsheet. Accordingly, M0, M2, and M3 were analyzed as categorical recorded assessment points and were not retrospectively assigned to fixed 24-, 48-, or 72-h windows, because doing so would introduce unsupported timing assumptions.

### 2.4. Laboratory Variables

The primary longitudinal laboratory family comprised PCT, CRP, WBC, platelet count, and serum creatinine. Hemoglobin was summarized descriptively but was not included among the five primary global tests. The verified reporting units were PCT (ng/mL), CRP (mg/L), WBC and platelets (×10^3^/µL), and creatinine (mg/dL).

Laboratory measurement units and analyzer platforms were verified retrospectively with the hospital laboratory using its reporting documentation and confirmation that the same equipment was used during the December 2024–December 2025 study period. White blood cell (WBC) and platelet counts were determined on a Sysmex XN-550 hematology analyzer (Sysmex Corporation, Kobe, Japan) and expressed as ×10^3^/µL (numerically equivalent to ×10^9^/L). The original platelet spreadsheet header stated “cells/mm^3^”; the hospital laboratory report clarified the stored reporting scale as ×10^3^/µL. C-reactive protein (CRP) was measured in mg/L using the Abbott CRP Vario reagent (turbidimetric assay) on the Abbott ARCHITECT c4000 system (Abbott Laboratories, Abbott Park, IL, USA). Serum creatinine was measured in mg/dL using the Abbott Creatinine2 reagent on the same ARCHITECT c4000 system, by a kinetic Jaffé method. Procalcitonin (PCT) was measured in ng/mL on a Roche cobas e 411 analyzer (Roche Diagnostics International Ltd., Rotkreuz, Switzerland) using electrochemiluminescence immunoassay (ECLIA). No retrospective numeric unit conversions or reagent-specific correction factors were applied to the frozen dataset.

### 2.5. Data Quality Control

Missing or non-numeric laboratory results were retained as missing and were not numerically imputed. Observations were not removed solely because of statistical extremeness. One M3 record contained an internally inconsistent adjacent-value pattern: platelet count was recorded as 3.05 and serum creatinine as 114.56, with the creatinine value duplicating the adjacent CRP value. Because the original laboratory report was unavailable and no defensible correction or column shift could be source-adjudicated, both the M3 platelet and M3 creatinine values for that episode were set to missing. No replacement value was imputed.

Extreme WBC measurements were retained when they were clinically and longitudinally coherent. Marked leukocytosis in episodes with documented chronic lymphocytic leukemia and acute myeloid leukemia was retained because the values were compatible with the recorded hematologic diagnoses and surrounding measurements. This decision was based on source coherence rather than statistical convenience.

### 2.6. Statistical Analysis

Continuous variables were summarized using mean and standard deviation and/or median and interquartile range, as appropriate. Measurement availability was reported separately at each assessment point. No complete-case restriction was imposed for the primary longitudinal models.

Separate random-intercept linear mixed-effects models were fitted for each of the five primary biomarkers, with assessment point entered as a categorical fixed effect and M0 as the reference category. Models were estimated by maximum likelihood. PCT and CRP were natural-log transformed because of marked right skewness and were reported as back-transformed geometric means, ratios of geometric means, and 95% confidence intervals. WBC, platelet count, and serum creatinine were analyzed on their recorded additive scales in the primary models.

The primary inferential test for each biomarker was the global two-degree-of-freedom Wald test of the categorical assessment-point effect. Family-wise error across the five primary global tests was controlled using the Holm procedure. Pairwise contrasts compared M2 versus M0, M3 versus M0, and M3 versus M2, with Holm adjustment applied to the three contrasts within each biomarker. Effect estimates and 95% confidence intervals were reported throughout.

Generalized estimating equations with an exchangeable working correlation structure and robust covariance estimation were used as model-specification sensitivity analyses. Additional natural-log mixed-effects models were fitted for positive-valued WBC, platelet count, and serum creatinine. Model convergence and residual diagnostics were reviewed; selected diagnostics are reported in Supplementary Table S6.

The five primary mixed-effects global tests were independently reproduced using Python 3.13.5 (Python Software Foundation; https://www.python.org/), statsmodels 0.14.6 (https://www.statsmodels.org/), and SciPy 1.17.0 (https://scipy.org/), with matching Wald statistics and *p* values in the frozen analytical dataset. The exact package versions of the original exploratory analysis were not separately archived.

No single-value imputation was performed. Baseline differences between episodes with and without M3 measurements were explored using standardized mean differences. These comparisons were used to assess whether a missing-completely-at-random mechanism appeared plausible; they cannot distinguish missing-at-random from missing-not-at-random mechanisms. At Reviewer 2’s request, we performed additional post hoc, exploratory two-sided group comparisons for the baseline missingness comparisons presented in the Results: Welch’s unequal-variance *t* test for age, Fisher’s exact test for sex, and the Mann–Whitney U test for baseline M0 biomarker values. Tests were restricted to observed values of each comparison variable. Because 15 tests were performed (three per biomarker), Holm-adjusted *p* values across these exploratory comparisons were also examined. Standardized mean differences were retained as the primary descriptors of baseline imbalance; these exploratory *p* values do not test or establish missingness mechanisms.

Exploratory interaction models evaluated time-by-anatomical-source phenotype and time-by-documented-vasopressor-use effects. Infection source was collapsed into pulmonary, abdominal/digestive, mixed, and other/unspecified categories. Interaction models included age and sex. False-discovery-rate adjustment across the five biomarker interaction tests was performed using the Benjamini–Hochberg procedure. Because vasopressor initiation timing was unavailable, vasopressor analyses were interpreted as exploratory associations only.

The analysis used all 100 admission episodes in the primary harmonizable three-assessment dataset. No post hoc observed-power calculation was used; inference is presented through effect estimates, 95% confidence intervals, prespecified multiplicity control, and sensitivity analyses. A mortality-stratified analysis was not performed because the source research spreadsheet contained a combined discharge/death date field without a definitive vital-status indicator, and the dedicated 28-day mortality field was not populated.

## 3. Results

### 3.1. Patient Characteristics and Baseline Comorbidities

The primary analytic cohort comprised 100 hospital admission episodes. Mean age was 66.7 years (SD 17.7), and median age was 71.0 years (IQR 58.0–79.0). Fifty-three episodes involved female patients and 47 male patients. Documented vasopressor use was present in 14 episodes. Infection-source categories were pulmonary in 20 episodes, abdominal/digestive in 23, mixed in 28, and other/unspecified in 29.

Source-record re-audit confirmed explicit sepsis wording in 100/100 episodes and identified no severe-infection-only records. Among the 100 admission episodes, the source comorbidity field recorded heart failure or NYHA-classified heart failure in 32, diabetes mellitus in 26, chronic kidney disease in 26, and dementia in 15. Seventeen episodes had source mentions of solid neoplasms or tumors, including historical diagnoses and lesions of unverified current activity; 7 included COPD or chronic bronchitis, 5 included autoimmune/connective-tissue conditions, 3 had recorded HIV infection, and 3 had hematologic malignancy or its history. These non-mutually exclusive counts are based on documented source wording; current disease activity, treatment-defined immune suppression, and immune competence were not uniformly ascertainable. Deidentified episode-level source-diagnosis and underlying-disease data are provided in Supplementary Table S7.

Availability of laboratory measurements decreased across successive assessment points. PCT was available in 94 episodes at M0, 79 at M2, and 59 at M3; CRP in 97, 89, and 64; WBC in 99, 93, and 71; platelet count in 99, 93, and 70 after quality control; and serum creatinine in 98, 88, and 66 after quality control (Table 1). Observed distributions by assessment point are reported in Supplementary Table S1, and measurement availability is visualized in Supplementary Figure S3.

### 3.2. Data-Quality Findings

No observations were excluded because they were statistically extreme. The internally inconsistent M3 platelet and creatinine values described above were set to missing because the source record could not support a defensible numerical correction. Clinically coherent extreme WBC values associated with recorded hematologic malignancies were retained. Details of these data-quality decisions are provided in Supplementary Table S5.

### 3.3. Primary Longitudinal Analyses

After Holm correction across the five primary global tests, significant assessment-point effects were observed for PCT, CRP, platelet count, and serum creatinine, whereas the full-cohort primary WBC model did not reach statistical significance (Table 2).

#### 3.3.1. Procalcitonin

PCT showed a significant overall assessment-point effect (Wald chi-square = 22.49, 2 df; Holm-adjusted *p* = 0.000052). The model-estimated geometric mean decreased from 0.925 ng/mL at M0 (95% CI 0.654–1.309) to 0.532 ng/mL at M2 (95% CI 0.366–0.773) and 0.306 ng/mL at M3 (95% CI 0.200–0.469). The GEE sensitivity analysis was concordant (*p* = 0.000070). Model-based estimates are displayed in Figure 2.

#### 3.3.2. C-Reactive Protein

CRP showed a significant overall assessment-point effect (Wald chi-square = 55.30, 2 df; Holm-adjusted *p* < 0.001). The model-estimated geometric mean decreased from 104.2 mg/L at M0 (95% CI 82.5–131.5) to 56.8 mg/L at M2 (95% CI 44.7–72.4) and 36.0 mg/L at M3 (95% CI 27.3–47.5). The GEE sensitivity analysis was concordant (*p* < 0.001). Model-based estimates are displayed in Figure 3.

#### 3.3.3. White Blood Cell Count

The full-cohort primary additive-scale WBC model did not demonstrate a statistically significant global assessment-point effect after correction across the five primary biomarkers (Wald chi-square = 5.20, 2 df; Holm-adjusted *p* = 0.0743). Model-estimated WBC values were 14.83 × 10^3^/µL at M0, 15.01 × 10^3^/µL at M2, and 12.33 × 10^3^/µL at M3. The GEE analysis was also non-significant (*p* = 0.147), whereas the logarithmic-scale sensitivity model was significant (*p* = 0.0229). Thus, WBC inference was sensitive to model specification; no consistent WBC trajectory was established by the prespecified primary analysis.

#### 3.3.4. Platelet Count

Platelet count showed a significant overall assessment-point effect on the additive scale (Wald chi-square = 18.08, 2 df; Holm-adjusted *p* = 0.000237), increasing from a model-estimated 223.4 × 10^3^/µL at M0 to 239.8 × 10^3^/µL at M2 and 268.6 × 10^3^/µL at M3. The GEE analysis supported an overall effect (*p* = 0.00229), whereas the logarithmic-scale sensitivity model was non-significant (*p* = 0.156). The platelet result was therefore dependent on analytical scale. The corresponding model-estimate display is provided in Supplementary Figure S1.

#### 3.3.5. Serum Creatinine

After the documented M3 data-quality exclusion, serum creatinine showed a significant overall assessment-point effect (Wald chi-square = 21.22, 2 df; Holm-adjusted *p* = 0.000074). Model-estimated serum creatinine decreased from 1.49 mg/dL at M0 (95% CI 1.30–1.68) to 1.27 mg/dL at M2 (95% CI 1.07–1.47) and 1.10 mg/dL at M3 (95% CI 0.88–1.31). The direction of change was concordant in the GEE analysis (*p* = 0.000064) and log-scale sensitivity model (*p* < 0.001). Model-based estimates are displayed in Figure 4.

### 3.4. Pairwise Contrasts

Pairwise assessment-point contrasts, their effect measures, 95% confidence intervals, and within-biomarker Holm-adjusted *p* values are provided in Table 3. The ratios for PCT and CRP compare back-transformed geometric means; additive differences apply to WBC, platelet count, and creatinine.

### 3.5. Robustness and Model Diagnostics

PCT and CRP showed concordant decreases in the primary mixed-effects and GEE analyses. Serum creatinine showed a concordant decrease across the additive mixed-effects, GEE, and logarithmic sensitivity specifications. Platelet count increased in the primary additive-scale and GEE analyses but not in the logarithmic sensitivity model. For WBC, the primary additive-scale model and GEE analysis were non-significant, whereas the logarithmic sensitivity model was significant. All primary mixed-effects models converged. Residual skewness was most pronounced for WBC (2.18) and serum creatinine (1.61), reinforcing the importance of sensitivity analyses and cautious interpretation (Supplementary Table S6). Full robustness results are summarized in Supplementary Table S2 and model diagnostics in Supplementary Table S6.

In a post hoc reviewer-requested sensitivity analysis, six episodes with explicitly recorded HIV infection (*n* = 3) or hematologic malignancy/history (*n* = 3) were excluded. Global assessment-point effects for PCT, CRP, platelet count, and serum creatinine remained significant after Holm correction. The WBC global test became significant in this restricted subset, underscoring its sensitivity to cohort composition rather than establishing a causal role for immune suppression (Supplementary Table S8).

### 3.6. Availability of the Final Recorded Assessment

M3 measurement availability was substantially lower than M0 availability for all primary biomarkers. Episodes with and without an M3 measurement differed in several baseline characteristics. Age standardized mean differences ranged from 0.315 to 0.500. Baseline CRP and baseline serum creatinine also differed between episodes with and without later measurements, with standardized mean differences of 0.523 and 0.482, respectively (Table 4). These imbalances raise concern that a missing-completely-at-random mechanism may be implausible; the available data cannot distinguish missing-at-random from missing-not-at-random mechanisms. Exploratory unadjusted tests identified lower *p* values for baseline CRP (*p* = 0.0092) and creatinine (*p* = 0.0119) according to later availability, as well as age in the creatinine comparison (*p* = 0.0323) and female sex in the PCT comparison (*p* = 0.0419); however, none of the 15 additional tests remained significant after Holm adjustment. These findings should be interpreted alongside the magnitude of standardized differences rather than as proof of any particular mechanism of missingness (Figure 5).

### 3.7. Exploratory Analyses

No time-by-anatomical-source interaction remained significant after false-discovery-rate correction. Adjusted interaction *p* values were 0.687 for PCT, 0.687 for CRP, 0.687 for WBC, 0.591 for platelet count, and 0.687 for serum creatinine. Full interaction results are reported in Supplementary Table S3.

Fourteen of the 100 episodes had documented vasopressor use. Exploratory time-by-vasopressor interaction analyses yielded false-discovery-rate-adjusted signals for CRP (*p* = 0.00835), platelet count (*p* = 0.00000279), and serum creatinine (*p* = 0.00859), but not for PCT or WBC. Because vasopressor initiation timing was unavailable and the exposed subgroup was small, these findings are hypothesis-generating associations and do not support temporal, predictive, causal, or septic-shock-onset interpretations. Full interaction results are reported in Supplementary Table S4.

## 4. Discussion

In this cohort, routine inflammatory, hematologic, and renal laboratory markers did not show a uniform longitudinal pattern. PCT and CRP decreased consistently across the primary mixed-effects and GEE analyses, while serum creatinine showed concordant decreases across additive, logarithmic, and GEE specifications. Platelet count increased in the additive-scale analyses but not after logarithmic transformation. The full-cohort primary WBC model was non-significant, and WBC inference was sensitive to analytical specification.

These findings describe cohort-level temporal differences that are not represented by a single baseline measurement. They do not imply that the evaluated markers represent interchangeable indicators of a single biological trajectory. The central contribution of the study is therefore the contrast in longitudinal behavior and statistical stability across routine markers from different biological domains, rather than the observation that any one biomarker changes during hospitalization.

The present cohort must be distinguished from both population-level epidemiology and clinically adjudicated prognostic cohorts. Romanian evidence provides useful context but not an interchangeable comparator. The large Bucharest hospital series included 12,089 coded or text-identified sepsis cases over 18 years, with outcome-oriented risk-factor analyses [5], whereas the Craiova investigation enrolled 184 individuals with community-acquired severe infections according to older SIRS/Sepsis-2-era definitions [6]. Varga et al. studied 138 patients with severe infections and emphasized mortality prediction using serial biomarkers [10]. By contrast, our outcome-free analysis characterizes marker-specific laboratory measurements across ordinal assessment points; differences in eligibility, infection severity, sampling schedules, and outcomes prevent direct comparison of effect magnitudes or mortality rates.

The declines in PCT and CRP were the most statistically concordant inflammatory findings in this cohort. Their direction is consistent with previous serial biomarker studies. Schupp et al. reported substantial temporal changes in CRP and PCT among 349 patients with sepsis or septic shock while also showing limited discrimination for 30-day mortality [8]. Orfanu et al. described distinct early biomarker kinetics in a prospective sepsis cohort [9]. Varga et al. evaluated serial PCT, CRP, and lactate in 138 patients with severe infections and focused on 30-day mortality prediction [10]. The present analysis addresses a different and narrower question: whether routinely measured markers from several biological domains show statistically concordant within-cohort changes across serial recorded assessments.

Accordingly, decreasing PCT or CRP in this study should not be interpreted as independent evidence of antimicrobial response, clinical recovery, microbiological clearance, or improved prognosis. Treatment exposure, microbiological response, organ dysfunction, and clinical outcomes were not characterized sufficiently to support those conclusions.

The biological distinction between PCT and CRP also cautions against treating their trajectories as interchangeable. CRP reflects hepatic acute-phase signaling and is modified by multiple noninfectious inflammatory processes, whereas PCT is regulated by different inflammatory pathways and can be influenced by renal dysfunction and infection-related factors [2,7,8,9]. Group-level geometric-mean decreases do not imply monotonic decline in every patient. Selection of which patients undergo later testing may further shape these averages. Without organism-level confirmation, antibiotic exposure timing, or clinical outcomes, a biomarker-only interpretation would be incomplete.

Serum creatinine showed a statistically concordant decrease across the primary additive-scale model, GEE analysis, and logarithmic sensitivity model after data-quality control. This finding should nevertheless not be equated with renal recovery. Takkavatakarn et al. identified multiple early serum-creatinine trajectories in critically ill patients with sepsis-associated acute kidney injury [14], and Liu et al. identified several distinct 72-h creatinine trajectories in a large sepsis cohort [15]. The current model estimates a cohort-average longitudinal pattern and does not identify patient-level renal phenotypes.

Serum creatinine is influenced by baseline renal function, fluid balance, renal replacement therapy, muscle mass, hemodynamic changes, and treatment exposure, several of which were unavailable for comprehensive adjustment. Acute kidney injury was not formally adjudicated using complete KDIGO criteria. The appropriate interpretation is therefore a concordant cohort-level decrease in recorded serum creatinine across the tested specifications, not proof of renal recovery.

Platelet count increased in the additive-scale mixed-effects and GEE analyses, but statistical significance was not retained after logarithmic transformation. This scale dependence is important because previous large studies have demonstrated multiple platelet trajectory phenotypes rather than a single universal pattern [16,17]. The current result should therefore be interpreted as an additive-scale cohort effect rather than a scale-invariant biological phenomenon. It cannot determine whether increasing counts reflect recovery from thrombocytopenia, reactive thrombocytosis, treatment effects, inflammatory change, or differential measurement availability.

WBC showed no significant global time effect in the full-cohort primary additive-scale model, and the GEE result was also non-significant; however, the logarithmic sensitivity model was significant. Residual diagnostics showed marked right-tail influence, and clinically coherent extreme leukocytosis associated with hematologic malignancy was intentionally retained. In the post hoc reviewer-requested sensitivity excluding six episodes with explicitly documented HIV infection or hematologic malignancy, the WBC global test became significant. Taken together, these findings argue against a single categorical interpretation of WBC and instead indicate substantial dependence on analytical specification and cohort composition, consistent with recent trajectory-based work [18].

This issue is clinically relevant in an infection-service cohort with diverse comorbidities. Six source records explicitly documented HIV infection or hematologic malignancy. The exploratory exclusion analysis showed a significant WBC time effect after restricting the sample, although WBC remained non-significant in the full-cohort primary model. Such a contrast should not be read as evidence that immune status caused a different trajectory: activity of malignant disease, immunosuppressive therapy, neutropenia, and timing of infection and treatment were incompletely captured. Rather, it illustrates how the analytic population can affect inferences about routinely measured cell counts. In contrast, the reported PCT/CRP/creatinine cohort-level patterns were more consistent across the examined specifications.

No time-by-source interaction remained significant after false-discovery-rate correction. This finding does not imply biological equivalence among infection sources. Peters-Sengers et al. demonstrated source-specific host-response differences in a larger and more extensively phenotyped sepsis cohort [13]. The present source variable required broad category collapse and the study was not powered for definitive interaction testing.

Exploratory time-by-vasopressor interaction signals for CRP, platelet count, and serum creatinine should be interpreted with even greater caution. Only 14 episodes had documented vasopressor use, and initiation timing relative to laboratory assessments was unavailable. Vasopressor use is also strongly related to illness severity and clinical management. These analyses therefore do not establish prediction of vasopressor requirement, septic shock, treatment response, or causality.

The progressive reduction in measurement availability at M3 is one of the principal limitations of the study. Baseline imbalances between episodes with and without M3 measurements suggest that a missing-completely-at-random mechanism may be implausible; however, the available data cannot determine whether missingness was missing at random or missing not at random. In routine care, repeat testing may depend on discharge, death, transfer, improvement, deterioration, or clinician decision, none of which was captured comprehensively.

Mixed-effects models allowed episodes with incomplete repeated measurements to contribute their observed data, but this does not eliminate bias if observation depends on unmeasured clinical status. Later model-estimated values may therefore reflect both within-episode biological change and differential measurement availability. This limitation is relevant particularly when interpreting M3 estimates.

The reviewer-requested post hoc Table 4 tests sharpen this distinction. Several unadjusted comparisons showed nominal evidence of imbalance, notably baseline CRP and serum creatinine, but no test remained significant after family-wise correction across 15 comparisons. This does not provide evidence for a missing-completely-at-random process: tests of observed baseline variables cannot resolve dependence of later observation on unmeasured clinical condition, transfer, death, or discharge. Standardized mean differences and clinical reasoning are therefore at least as important as *p* values. Methods literature emphasizes that the validity of incomplete-data inference depends on assumptions about unobserved outcomes that cannot be verified from the observed data alone [19].

Methodological strengths include explicit multiplicity control across five primary global tests, within-biomarker adjustment of pairwise contrasts, sensitivity analyses using GEE and alternative outcome scales, and transparent data-quality decisions that distinguished clinically plausible extreme observations from a non-adjudicable source-record inconsistency. The analyses also retained incomplete longitudinal records rather than restricting inference to complete cases.

Several limitations constrain interpretation. First, the primary cohort was modest in size and derived from a single harmonizable three-assessment source dataset; a second dataset was not pooled because laboratory harmonization could not be established and M3 measurements were unavailable, which may limit generalizability. Second, although all 100 source records explicitly contained a clinical sepsis label, the diagnosing service was confirmed as infectious diseases but individual diagnosing clinicians and episode-specific diagnostic algorithms were not retained, and complete SOFA components were unavailable; retrospective Sepsis-3 adjudication was therefore not possible. Third, exact elapsed times between M0, M2, and M3 were not recorded, so the analysis cannot be interpreted as fixed 24-, 48-, or 72-h kinetics. Fourth, systematic microbiological data and recovered pathogens were not retained in File1. Fifth, the source extract did not systematically capture treatment-defined immune status, preventing definitive immunocompetent-versus-immunocompromised classification. Sixth, sufficiently complete clinical outcomes, including definitive in-hospital vital status and dedicated 28-day mortality, were unavailable, precluding survival-stratified biomarker analyses. Seventh, later measurement availability was incomplete and its mechanism could not be established. Eighth, definitive patient-level linkage was unavailable, so repeated admissions by the same individual could not be ruled out completely. Ninth, the deidentified extract did not store per-result analyzer, reagent lot, or complete laboratory provenance data, although units, platforms, and assay methods were confirmed retrospectively with the hospital laboratory. Finally, exploratory source and vasopressor analyses were limited by small subgroups and incomplete temporal information.

Laboratory reporting units and analyzer platforms were retrospectively confirmed with the hospital laboratory, including the original platelet header discrepancy. Nevertheless, analyte-specific sampling timestamps, per-record analyzer identifiers, and reagent lot/batch information were not available in the deidentified research extract. Although the confirmed units make absolute reporting possible, the present observational comparisons do not establish clinical thresholds or inter-laboratory interchangeability.

The findings support marker-specific interpretation of serial routine laboratory measurements rather than a single generalized concept of biomarker improvement. For clinical use, the present estimates cannot specify treatment thresholds or time-dependent decision rules: M2/M3 were ordinal assessments without confirmed elapsed-hour windows, and clinical deterioration, interventions, and outcomes were not systematically recorded. For future research, prospectively scheduled sampling, linked laboratory-system timestamps, complete organ-dysfunction measures, pathogen ascertainment, immunosuppressive treatment history, and survival follow-up would permit direct comparisons of biological and prognostic trajectories. Larger multicenter cohorts with adequate repeated observations could also support patient-level trajectory modeling, missingness sensitivity analyses, and more defensible transportability assessment. In this context, the contribution of the present work is transparency about the discordance between different biomarker families and the fragility of some inferences, rather than a claim of novel diagnostic or prognostic performance.

## 5. Conclusions

In adults hospitalized with clinically recorded sepsis, serial routine laboratory markers did not evolve concordantly across the recorded assessment points. PCT and CRP showed consistent decreases across the primary mixed-effects and GEE analyses, and serum creatinine showed concordant decreases across additive, logarithmic, and GEE specifications. Platelet findings were scale-dependent, while the full-cohort WBC model did not show a significant primary time effect and remained sensitive to cohort/model specification. These findings describe cohort-level laboratory patterns and should not be interpreted as evidence of treatment response, organ recovery, microbiological clearance, or prognosis. Confirmation in prospectively characterized cohorts with standardized sampling times, complete organ-dysfunction data, microbiological characterization, verified laboratory metadata, and clinically informative follow-up is required.

## Figures and Tables

**Figure 1:**
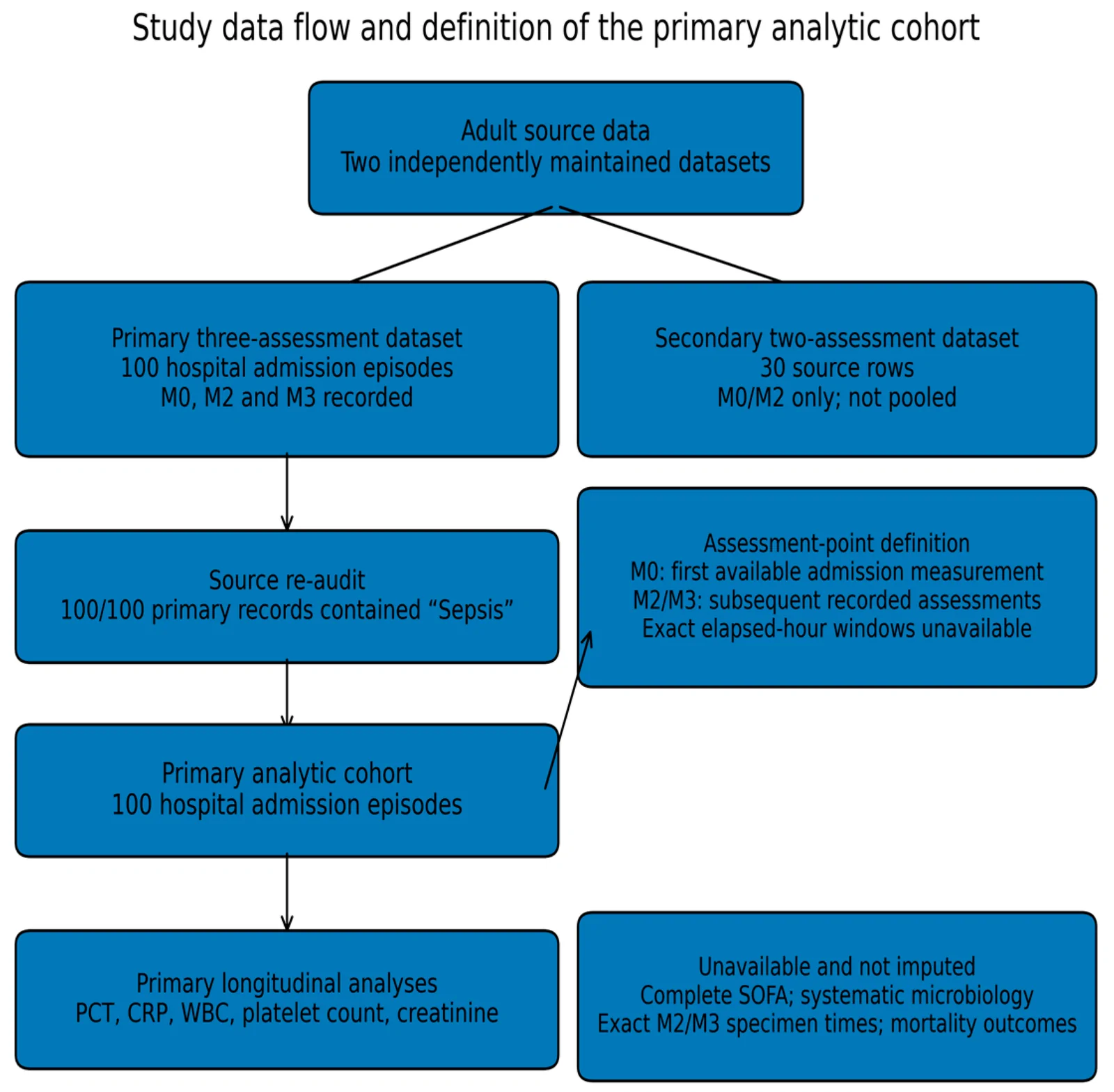
Study data flow and definition of the primary analytic cohort. The primary three-assessment source dataset contained 100 hospital admission episodes; all 100 source records explicitly contained the term “Sepsis”. A secondary two-assessment dataset contained 30 source rows but was not pooled because laboratory harmonization could not be established with sufficient confidence and M3 measurements were unavailable. M0 denotes the first available admission measurement; M2 and M3 are subsequent ordered recorded assessments for which exact elapsed-hour windows were not retained. Unique-patient count could not be confirmed from deidentified data. One M3 platelet value and one M3 creatinine value were set to missing after data-quality review; no numerical correction was imputed. Abbreviations: PCT, procalcitonin; CRP, C-reactive protein; WBC, white blood cell count; SOFA, Sequential Organ Failure Assessment.

**Figure 2:**
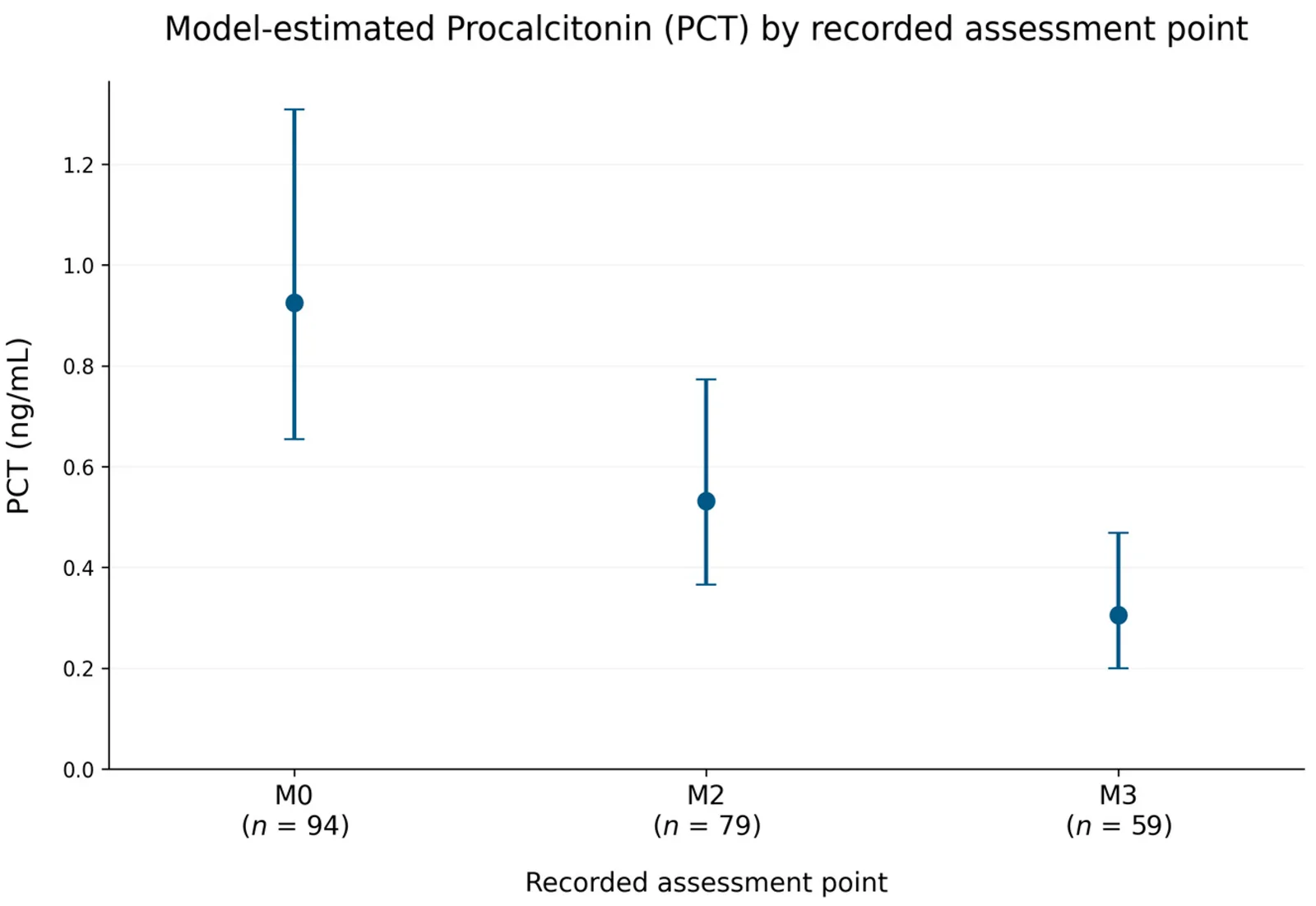
Model-estimated procalcitonin concentrations across serial recorded assessment points. Points represent back-transformed geometric means from the primary random-intercept linear mixed-effects model; error bars represent 95% confidence intervals. Observed sample sizes (*n*) were 94 at M0, 79 at M2, and 59 at M3.

**Figure 3:**
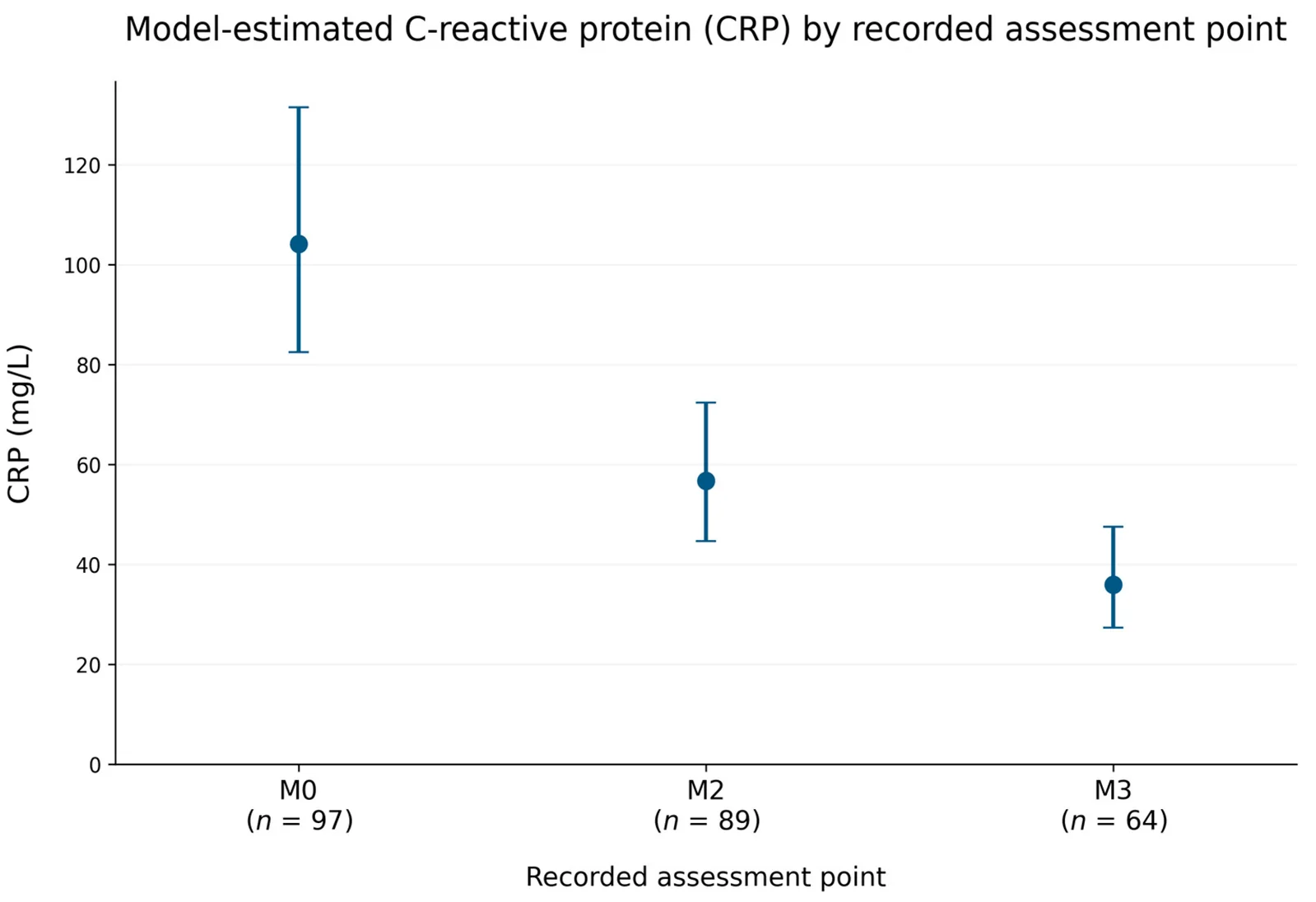
Model-estimated C-reactive protein concentrations across serial recorded assessment points. Points represent back-transformed geometric means from the primary random-intercept linear mixed-effects model; error bars represent 95% confidence intervals. Observed sample sizes (*n*) were 97 at M0, 89 at M2, and 64 at M3.

**Figure 4:**
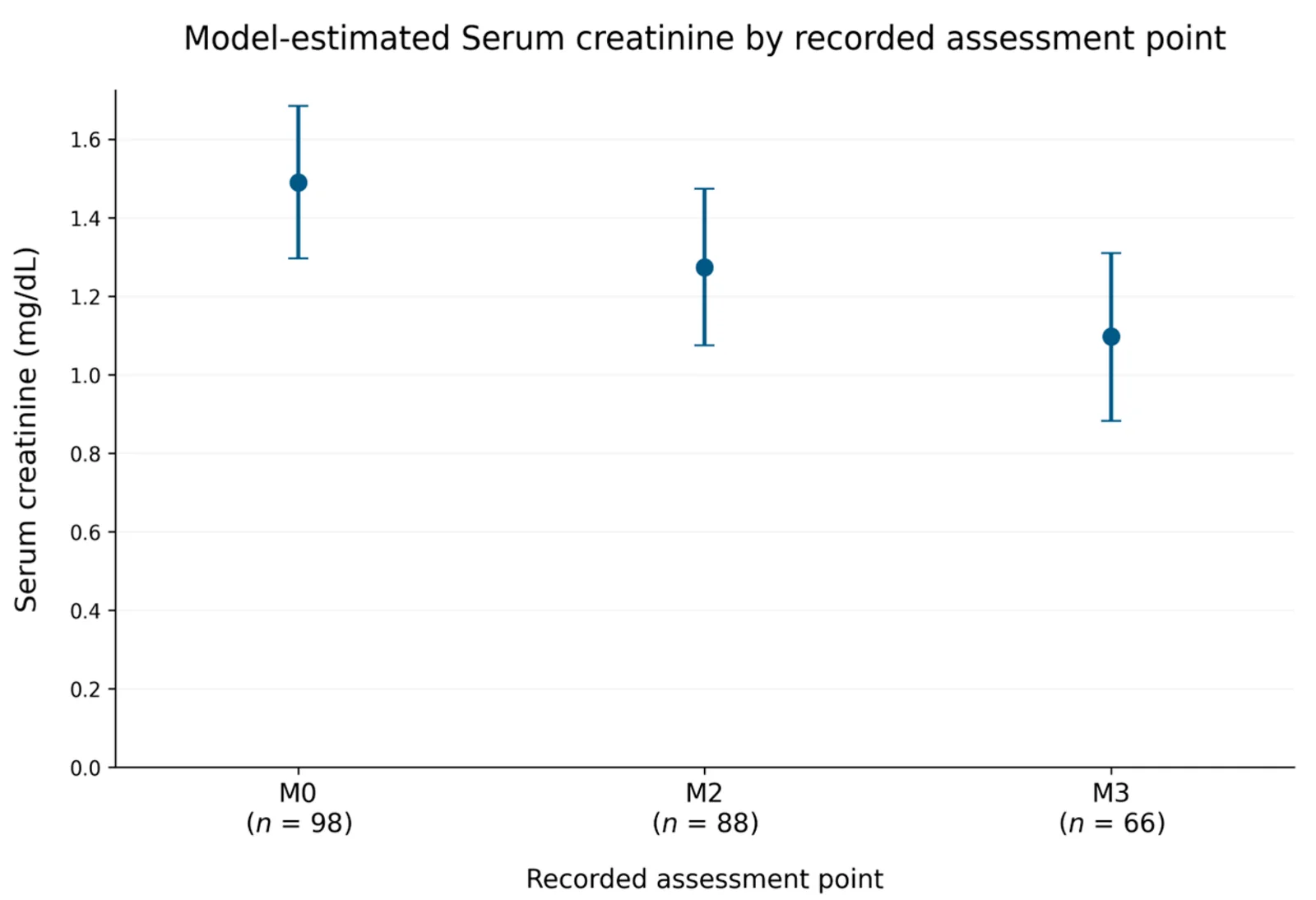
Model-estimated serum creatinine across serial recorded assessment points after data-quality control. Points represent model-estimated means from the primary random-intercept linear mixed-effects model; error bars represent 95% confidence intervals. Observed sample sizes (*n*) were 98 at M0, 88 at M2, and 66 at M3.

**Figure 5:**
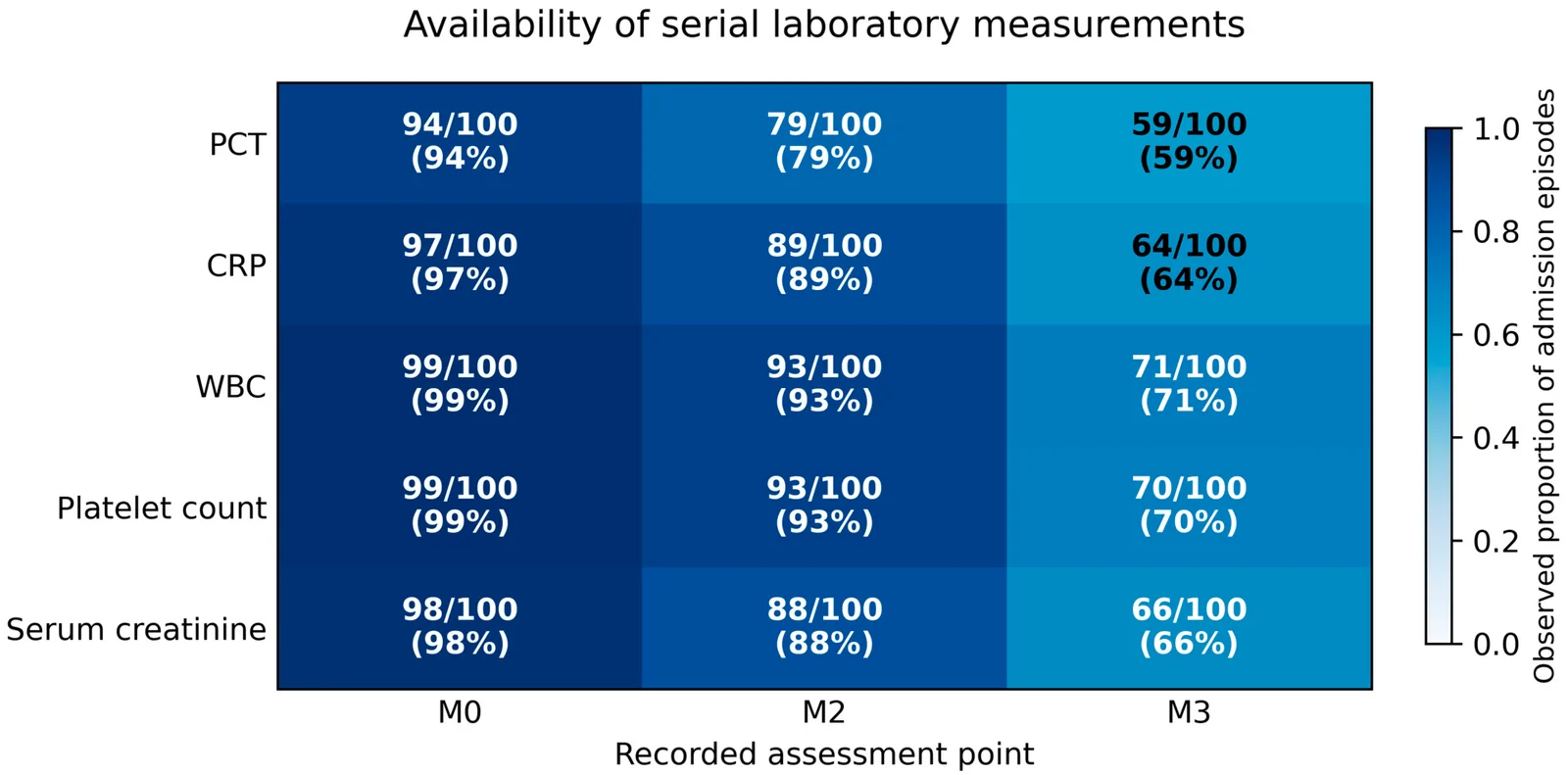
Availability of primary laboratory measurements across the three ordered recorded assessment points (M0, M2, M3). Each cell gives the observed number of episodes (denominator = 100) and percentage. An unavailable laboratory result does not indicate zero concentration or an adverse outcome; exact sampling times and reasons for non-observation were not retained.

**Table 1:** Cohort characteristics and serial measurement availability.

Characteristic/Biomarker	M0	M2	M3	Value/All 3
Cohort characteristics				
Hospital admission episodes, *n*	100			
Age, mean (SD), years	66.7 (17.7)			
Age, median (IQR), years	71.0 (58.0–79.0)			
Female, *n* (%)	53 (53%)			
Male, *n* (%)	47 (47%)			
Documented vasopressor use, *n* (%)	14 (14%)			
Pulmonary source, *n* (%)	20 (20%)			
Abdominal/digestive source, *n* (%)	23 (23%)			
Mixed source, *n* (%)	28 (28%)			
Other/unspecified source, *n* (%)	29 (29%)			
Serial measurement availability				
PCT	94	79	59	56 (56%)
CRP	97	89	64	58 (58%)
WBC	99	93	71	70 (70%)
Platelet count	99	93	70	69 (69%)
Serum creatinine	98	88	66	60 (60%)
Hemoglobin	99	93	71	70 (70%)

M0 was the first available measurement on admission. M2 and M3 were treated as ordered recorded assessment points without source-verified exact-hour intervals. Platelet and creatinine M3 counts reflect the documented data-quality exclusions.

**Table 2:** Primary longitudinal mixed-effects models and model-estimated values.

Biomarker	Primary Scale	Observations	Episodes	M0 Estimate (95% CI)	M2 Estimate (95% CI)	M3 Estimate (95% CI)	Wald Chi-Square	df	Holm-Adjusted *p*
PCT	ln	232	95	0.925 (0.654–1.309)	0.532 (0.366–0.773)	0.306 (0.200–0.469)	22.49	2	0.000052
CRP	ln	250	99	104.2 (82.5–131.5)	56.8 (44.7–72.4)	36.0 (27.3–47.5)	55.30	2	<0.000001
WBC	additive (×10^3^/µL)	263	99	14.83 (10.89–18.76)	15.01 (11.04–18.98)	12.33 (8.21–16.44)	5.20	2	0.0743
Platelet count	additive (×10^3^/µL)	262	99	223.4 (195.8–250.9)	239.8 (211.9–267.7)	268.6 (239.3–298.0)	18.08	2	0.000237
Serum creatinine	mg/dL, additive	252	99	1.490 (1.296–1.685)	1.274 (1.075–1.474)	1.097 (0.883–1.310)	21.22	2	0.000074

PCT and CRP estimates are back-transformed geometric means. WBC, platelet count, and serum creatinine are model-estimated means on their prespecified additive scales. Units: PCT ng/mL, CRP mg/L, WBC and platelet count ×10^3^/µL, and creatinine mg/dL.

**Table 3:** Pairwise contrasts between recorded assessment points.

Biomarker	Contrast	Effect Measure	Estimate	95% CI	Raw *p*	Holm-Adjusted *p*
PCT	M2 vs. M0	Ratio of geometric means	0.575	0.379–0.870	0.00893	0.0179
PCT	M3 vs. M0	Ratio of geometric means	0.331	0.208–0.525	0.00000272	0.00000817
PCT	M3 vs. M2	Ratio of geometric means	0.575	0.358–0.924	0.0222	0.0222
CRP	M2 vs. M0	Ratio of geometric means	0.546	0.423–0.704	0.00000309	0.00000617
CRP	M3 vs. M0	Ratio of geometric means	0.346	0.259–0.461	<0.000001	<0.000001
CRP	M3 vs. M2	Ratio of geometric means	0.634	0.474–0.848	0.00211	0.00211
WBC	M2 vs. M0	Mean difference (×10^3^/µL)	+0.184	−2.068 to 2.436	0.873	0.873
WBC	M3 vs. M0	Mean difference (×10^3^/µL)	−2.503	−4.999 to −0.007	0.0493	0.108
WBC	M3 vs. M2	Mean difference (×10^3^/µL)	−2.687	−5.196 to −0.177	0.0359	0.108
Platelet count	M2 vs. M0	Mean difference (×10^3^/µL)	+16.39	−2.37 to 35.14	0.0868	0.0868
Platelet count	M3 vs. M0	Mean difference (×10^3^/µL)	+45.25	24.37 to 66.12	0.0000216	0.0000647
Platelet count	M3 vs. M2	Mean difference (×10^3^/µL)	+28.86	7.85 to 49.86	0.00708	0.0142
Serum creatinine	M2 vs. M0	Mean difference, mg/dL	−0.216	−0.369 to −0.063	0.00570	0.0114
Serum creatinine	M3 vs. M0	Mean difference, mg/dL	−0.394	−0.565 to −0.223	0.00000643	0.0000193
Serum creatinine	M3 vs. M2	Mean difference, mg/dL	−0.178	−0.352 to −0.003	0.0459	0.0459

Within each biomarker, the three pairwise contrasts were adjusted using the Holm procedure. For PCT and CRP, ratios below 1 indicate lower values at the later assessment; mean differences are later minus earlier assessment.

**Table 4:** Baseline characteristics by availability of an M3 measurement, with exploratory post hoc group comparisons.

Biomarker	M3 Present/Absent, *n*	Age Present/Absent, Mean y	Age SMD	Age *p*	Female Present/Absent, %	Sex SMD	Sex *p*	M0 SMD	M0 *p*
PCT	59/41	69.5/62.6	0.398	0.060	44.1/65.9	−0.438	0.042	0.294	0.057
CRP	64/36	69.2/62.1	0.412	0.067	48.4/61.1	−0.255	0.297	0.523	0.009
WBC	71/29	68.6/62.0	0.379	0.115	50.7/58.6	−0.159	0.514	0.282	0.123
Platelet count	70/30	68.3/62.8	0.315	0.185	51.4/56.7	−0.105	0.667	−0.212	0.336
Serum creatinine	66/34	69.6/61.0	0.500	0.032	50.0/58.8	−0.177	0.526	0.482	0.012

SMD = standardized mean difference; present/absent refers to availability of the M3 measurement for the specified biomarker. Age: Welch unequal-variance *t* test; female sex: two-sided Fisher exact test; M0 biomarker: two-sided Mann–Whitney U test using available baseline observations. Unadjusted two-sided *p* values are displayed for transparency (15 exploratory tests); none remained significant following Holm correction across all 15 comparisons. The tests were added at peer-review and were not part of the prespecified primary analysis. Non-significant *p* values do not demonstrate that missingness is completely at random.

## Data Availability

The underlying patient-level clinical datasets are not publicly available due to patient-confidentiality, ethical, and institutional data-protection restrictions. Requests for access may be directed to the corresponding author and are subject to authorization by the data-holding institution.

## Supplementary Materials

The following supporting information can be downloaded at: https://www.mdpi.com/article/10.3390/jcm15218246/s1, Table S1, Observed biomarker distributions by recorded assessment point; Table S2, Robustness and scale-sensitivity analyses; Table S3, Exploratory time-by-anatomical-source interactions; Table S4, Exploratory time-by-documented-vasopressor-use interactions; Table S5, Data-quality decisions relevant to inferential analyses; Table S6, Primary mixed-effects model diagnostics; Table S7, Deidentified admission-episode-level source diagnoses, underlying diseases and availability of SOFA, microbiology and outcome data; Table S8, Post hoc sensitivity excluding episodes with recorded HIV infection or hematologic malignancy/history; Figure S1, Model-estimated platelet count across recorded assessment points; Figure S2, Model-estimated white blood cell count across recorded assessment points.

## References

[1] Singer M., Deutschman C.S., Seymour C.W., Shankar-Hari M., Annane D., Bauer M., Bellomo R., Bernard G.R., Chiche J.D., Coopersmith C.M. (2016). The Third International Consensus Definitions for Sepsis and Septic Shock (Sepsis-3). JAMA.

[2] van der Poll T., van de Veerdonk F.L., Scicluna B.P., Netea M.G. (2017). The immunopathology of sepsis and potential therapeutic targets. Nat. Rev. Immunol..

[3] Rudd K.E., Johnson S.C., Agesa K.M., Shackelford K.A., Tsoi D., Kievlan D.R., Colombara D.V., Ikuta K.S., Kissoon N., Finfer S. (2020). Global, regional, and national sepsis incidence and mortality, 1990–2017: Analysis for the Global Burden of Disease Study. Lancet.

[4] GBD 2021 Global Sepsis Collaborators (2025). Global, regional, and national sepsis incidence and mortality, 1990–2021: A systematic analysis. Lancet Glob. Health.

[5] Mușat F., Păduraru D.N., Bolocan A., Palcău C.-A., Bunea A.-A., Ion D., Andronic O. (2025). Sepsis Burden in a Major Romanian Emergency Center—An 18-Year Retrospective Analysis of Mortality and Risk Factors. Medicina.

[6] Grigorescu A., Dumitrescu F., Dorobantu S., Dragos A., Pirvu A., Roskanovic M., Streata I., Ioana M., Netea M.G., on behalf of the FUSE study (2025). An Epidemiological Survey of Sepsis in a Tertiary Academic Hospital from Southwestern Romania. Medicina.

[7] Bourika V., Rekoumi E.A., Giamarellos-Bourboulis E.J. (2025). Biomarkers to guide sepsis management. Ann. Intensive Care.

[8] Schupp T., Weidner K., Rusnak J., Jawhar S., Forner J., Dulatahu F., Dudda J., Brueck L.M., Hoffmann U., Bertsch T. (2024). C-reactive protein and procalcitonin during course of sepsis and septic shock. Ir. J. Med. Sci..

[9] Orfanu A., Arama V., Popescu C., Tiliscan C., Streinu-Cercel A., Arama S.S. (2020). The Dynamical Assessment of Inflammatory Biomarkers in Predicting the Outcome of Septic Patients and the Response to Antimicrobial Therapy. J. Crit. Care Med..

[10] Varga N.I., Benea A.T., Suba M.I., Bota A.V., Avram C.R., Boru C., Dragomir T.L., Prisca M., Tanasescu S., Susan M. (2024). Predicting Mortality in Sepsis: The Role of Dynamic Biomarker Changes and Clinical Scores-A Retrospective Cohort Study. Diagnostics.

[11] Xu P., Lou J., Kong H., Li J., Xiang Z., Zhu X., Cui S., Huang N., Xu S., Le X. (2026). Dynamic biomarker profiling and phenotyping in burn sepsis: A retrospective cohort study using growth mixture modeling. Front. Cell. Infect. Microbiol..

[12] Turcato G., Zaboli A., Filippi L., Maggi M., Bionda A.E., Lucente F., Caregnato A., Milazzo D., Ferretto P., Ghiadoni L. (2026). Temporal Dissociation Between Intravascular Albumin Mass and Transcapillary Escape Dynamics in Sepsis: A Longitudinal Characterization of Albumin Homeostasis Using Routine Laboratory Parameters. J. Clin. Med..

[13] Peters-Sengers H., Butler J.M., Uhel F., Schultz M.J., Bonten M.J., Cremer O.L., Scicluna B.P., van Vught L.A., van der Poll T., MARS Consortium (2022). Source-specific host response and outcomes in critically ill patients with sepsis: A prospective cohort study. Intensive Care Med..

[14] Takkavatakarn K., Oh W., Chan L., Hofer I., Shawwa K., Kraft M., Shah N., Kohli-Seth R., Nadkarni G.N., Sakhuja A. (2024). Machine learning derived serum creatinine trajectories in acute kidney injury in critically ill patients with sepsis. Crit. Care.

[15] Liu D., Zhang Z., Qin A., Li X., Zhao J., Jiang H., Zhao Y., Chen Y., Peng H. (2026). Trajectory of early creatinine change is associated with the prognosis of sepsis patients. BMC Infect. Dis..

[16] Wang K., Lu D., Wang F. (2024). Subphenotypes of platelet count trajectories in sepsis from multi-center ICU data. Sci. Rep..

[17] Zhang X., Cai J., Ni C., Ma Y., Wu Y., Yang H., Ye F. (2026). Group-based trajectory modeling of platelet dynamics in sepsis: From phenotypic identification to the exploration of prognostic mediators. Front. Med..

[18] Miao S., Liu Y., Li M., Yan J. (2025). Clinical subtypes identification and feature recognition of sepsis leukocyte trajectories based on machine learning. Sci. Rep..

[19] Little R.J., D’Agostino R., Cohen M.L., Dickersin K., Emerson S.S., Farrar J.T., Frangakis C., Hogan J.W., Molenberghs G., Murphy S.A. (2012). The Prevention and Treatment of Missing Data in Clinical Trials. N. Engl. J. Med..

