# Supplementary material for: Serial Changes in Routine Inflammatory, Hematologic, and Renal Laboratory Markers in Adults Hospitalized with Clinically Recorded Sepsis: A Longitudinal Observational Cohort Study
Source: J Clin Med. 2026 Oct 23;15(21):8246. doi: 10.3390/jcm15218246 (PMC13763679; doi:10.3390/jcm15218246)
Supplement: Supplementary file 1 [file jcm-15-08246-s001.zip › Supplementary_Materials_Tables_S1-S6_Figures_S1-S2.pdf]

Supplementary Materials

For: Serial Changes in Routine Inflammatory, Hematologic, and Renal Laboratory Markers in Adults Hospitalized with Clinically Recorded Sepsis: A Longitudinal Observational Cohort Study

Andra Rotea[1,†]; Bogdan Rotea[1,2,4,†,*]; Mădălina Suba[1,3,4]; Ovidiu Roșca[3,5]; Bogdan Cerbu[1,2,6]; Ileana Enătescu[2,6]; Mirabela Dima[2,6]; Emil Radu Iacob[2,7]; Adrian Gluhovschi[8]; Daniela Iacob[2,6]

Supplementary Tables S1–S6 and Supplementary Figures S1–S2 accompany the manuscript in this document. The earlier measurement-availability figure has been promoted to main Figure 5. Supplementary Table S7 is a separate deidentified episode-level source-diagnosis/underlying-disease spreadsheet without hospital record numbers, initials, exact ages, sex, or care dates, and Supplementary Table S8 contains the post hoc reviewer-requested sensitivity analysis.

Supplementary Table S1. Observed biomarker distributions by recorded assessment point.

Biomarker	Assessment	N	Mean	SD	Median	Q1	Q3	Min	Max
PCT	M0	94	4.572	11.890	0.755	0.233	3.143	0.03	92.85
PCT	M2	79	2.398	6.844	0.430	0.170	1.220	0.02	42.51
PCT	M3	59	4.573	18.460	0.230	0.120	0.610	0.01	135.26
CRP	M0	97	151.675	108.541	137.470	63.770	227.220	6.20	512.90
CRP	M2	89	99.912	87.644	77.820	29.600	147.780	0.48	356.26
CRP	M3	64	79.942	96.300	47.945	15.333	105.345	2.46	507.57
WBC	M0	99	14.828	16.840	11.130	8.080	16.000	0.93	138.06
WBC	M2	93	15.403	23.205	10.950	7.740	15.630	1.47	171.93
WBC	M3	71	13.905	22.869	10.380	7.415	13.430	1.05	193.38
Platelet count	M0	99	223.384	124.704	204.0	148.0	279.0	10	705
Platelet count	M2	93	238.806	140.700	218.0	147.0	299.0	12	730
Platelet count	M3	70	260.659	165.645	246.5	144.25	351.25	10	1007
Serum creatinine	M0	98	1.479	1.109	1.165	0.773	1.810	0.40	8.35
Serum creatinine	M2	88	1.299	0.942	1.010	0.678	1.553	0.38	5.50
Serum creatinine	M3	66	1.196	0.943	0.900	0.650	1.228	0.21	6.53
Hemoglobin	M0	99	11.107	2.358	11.2	9.2	12.75	5.6	17.3
Hemoglobin	M2	93	10.900	2.237	10.9	9.1	12.5	5.7	15.5
Hemoglobin	M3	71	10.520	2.115	10.5	8.8	12.05	6.5	15.3

Supplementary Table S2. Robustness and scale-sensitivity analyses.

Biomarker	Primary Holm-adjusted p	GEE p	Log-scale sensitivity p	Interpretation
PCT	0.000052	0.000070	Not applicable	Primary mixed-effects and GEE analyses concordant
CRP	<0.000001	<0.000001	Not applicable	Primary mixed-effects and GEE analyses concordant
Serum creatinine	0.000074	0.000064	<0.000001	Direction concordant across additive, GEE, and log specifications
Platelet count	0.000237	0.00229	0.156	Inference depends on outcome scale
WBC	0.0743	0.147	0.0229	Primary/GEE non-significant; log-scale sensitivity significant

Supplementary Table S3. Exploratory time-by-anatomical-source interactions.

Biomarker	Scale	Observations	Episodes	Interaction chi-square	df	Raw p	BH-FDR p
PCT	ln	232	95	5.436	6	0.489	0.687
CRP	ln	250	99	4.466	6	0.614	0.687
WBC	identity	263	99	4.181	6	0.652	0.687
Platelet count	identity	262	99	10.156	6	0.118	0.591
Serum creatinine	identity	252	99	3.924	6	0.687	0.687

Supplementary Table S4. Exploratory time-by-documented-vasopressor-use interactions.

Biomarker	Scale	Observations	Episodes	Interaction chi-square	df	Raw p	BH-FDR p
PCT	ln	232	95	2.179	2	0.336	0.336
CRP	ln	250	99	11.404	2	0.00334	0.00835
WBC	identity	263	99	2.659	2	0.265	0.331
Platelet count	identity	262	99	28.798	2	<0.000001	0.00000279
Serum creatinine	identity	252	99	10.536	2	0.00515	0.00859

These analyses are exploratory. Documented vasopressor use was present in 14 of 100 episodes, and initiation timing relative to laboratory measurements was unavailable.

Supplementary Table S5. Data-quality decisions relevant to inferential analyses.

Study ID	Assessment	Variable	Raw value(s)	Decision	Rationale
AS-0031	M3	Platelet count	3.05	Set missing	Internally inconsistent adjacent-value pattern; no defensible source-adjudicated correction.
AS-0031	M3	Serum creatinine	114.56	Set missing	Duplicated the adjacent CRP value and was incompatible with the surrounding row; original laboratory report unavailable.
AS-0002	M0-M3	WBC	138.06 -> 171.93 -> 193.38	Retained	Extreme leukocytosis clinically coherent with documented chronic lymphocytic leukemia and longitudinal counts.
AS-0078	M0-M3	WBC	100.52 -> 155.67 -> 56.69	Retained	Extreme leukocytosis clinically coherent with documented acute myeloid leukemia and longitudinal counts.

Supplementary Table S6. Primary mixed-effects model diagnostics.

Biomarker	Scale	Obs.	N	Conv.	Optimizer	RI var.	Residual var.	ICC	Skew
PCT	ln	232	95	Yes	bfgs	1.0923	1.8589	0.3701	0.7617
CRP	ln	250	99	Yes	bfgs	0.6168	0.7629	0.4470	-0.6022
WBC	identity	263	99	Yes	lbfgs	337.262	61.794	0.8452	2.1818
Platelet count	identity	262	99	Yes	lbfgs	15270.54	4293.57	0.7805	0.1320
Serum creatinine	identity	252	99	Yes	lbfgs	0.6993	0.2733	0.7190	1.6101

Supplementary Figures

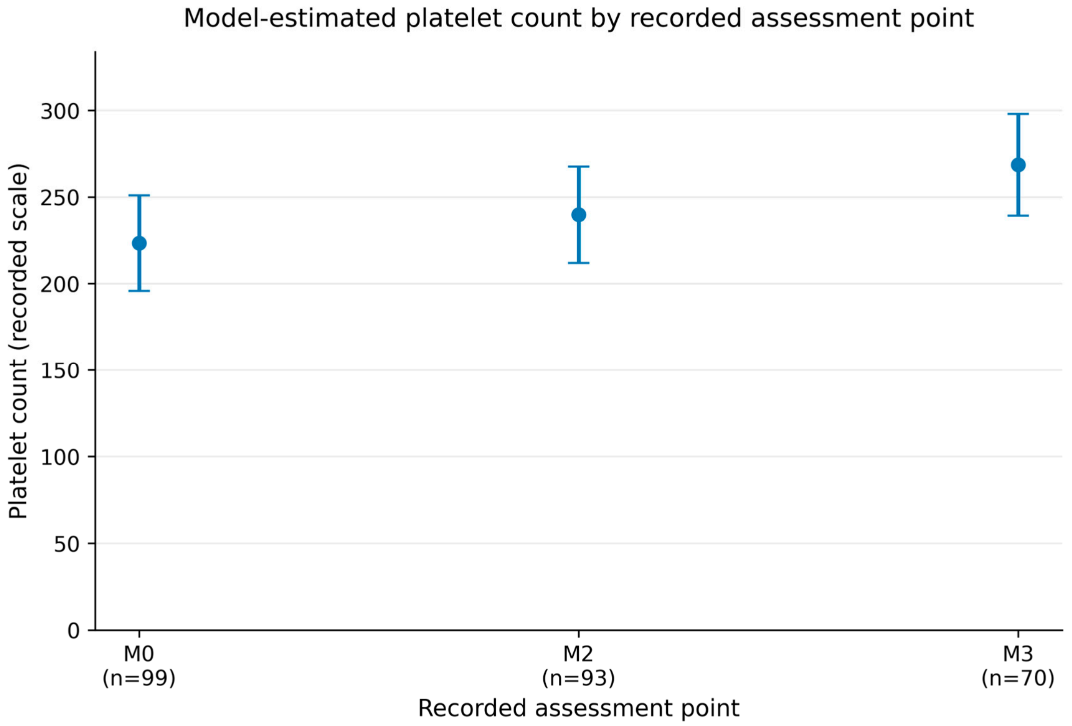

Supplementary Figure S1. Model-estimated platelet values across serial recorded assessment points. The additive-scale mixed-effects model and GEE analysis supported an increase, whereas the logarithmic sensitivity model was non-significant. Values are shown in ×10³/µL (equivalent numerically to ×10⁹/L), as retrospectively confirmed by the hospital laboratory.

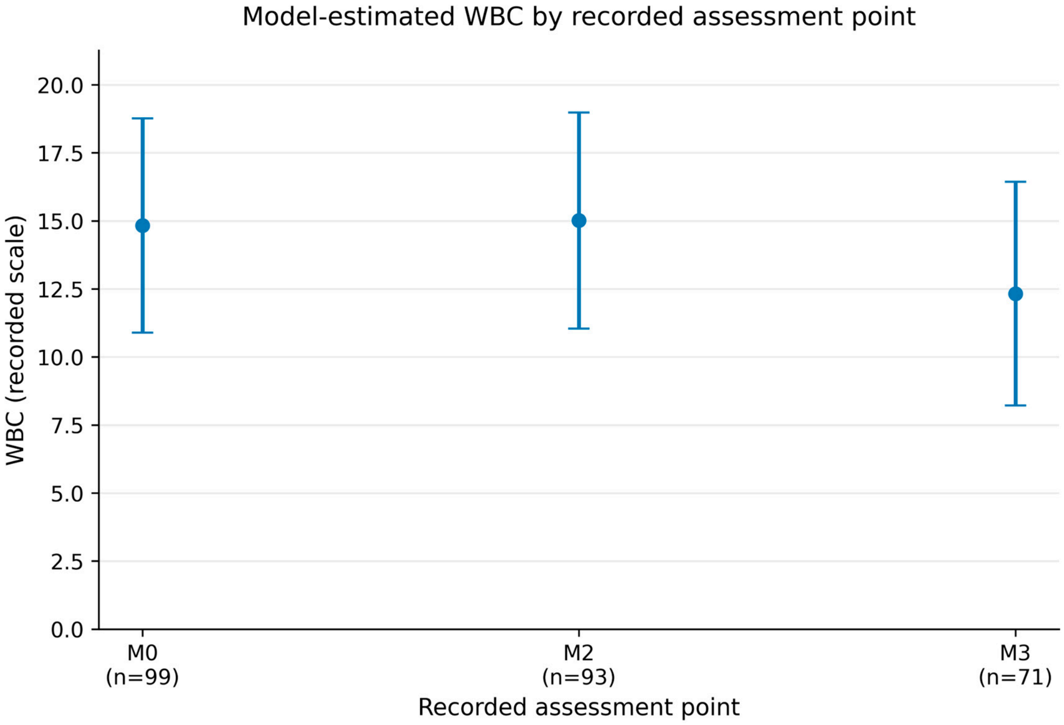

Supplementary Figure S2. Model-estimated WBC values across serial recorded assessment points. The primary additive-scale mixed-effects model and GEE analysis were non-significant, whereas the logarithmic sensitivity model was significant. Values are shown in ×10³/µL (equivalent numerically to ×10⁹/L), as retrospectively confirmed by the hospital laboratory.
